# Hospitals of the North-West

**Published:** 1891-11-14

**Authors:** 


### Hospitals of the North-West.

By our Special Commissioner.

### I.—WINNIPEG AND MEDICINE HAT.

It is curious as we travel through the Dominion of Canada to note how a great railway affects all interests, and promotes the establishment of institutions never before found in a community. Of course, in a new country development is necessarily slow; but it is a remarkable fact that in the district known as the Great North-West, which extends from Winnipeg to Vancouver—a distance of 1,500 miles by railway—there are only three hospitals, which are situated at Winnipeg, Medicine Hat, and Vancouver respectively. This journey occupies three days and nights. Medicine Hat receives patients from the Rockies, and all places as far west as Banff (560 miles from Vancouver) have to be sent to this institution. Cases of accident occurring in the Rocky and Selkirk Mountains, say between Banff and Golden, or Moberley, appear to be taken over to Vancouver or back to Medicine Hat, as the train service may best suit at the moment. Practically, Medicine Hat is more often selected for such cases. So it will be seen that each hospital supplies a radius of something like 250 miles; but the number of beds is not great, owing to the population being sparse and scanty, though much evidence exists that the inhabitants will enormously increase before many years have passed.

We will now take the three hospitals in order, giving a brief account of each.

### Winnipeg General Hospital.

This hospital consists of separate buildings containing (*a*) administration and general wards; (*b*) a Nurses' Home; (*c*) a Maternity Hospital. The report does not state the number of beds, or how these beds were distributed, but we believe the hospital to contain somewhere about 60 beds in all. The buildings are, on the whole, well adapted for their purpose, but it is certainly a matter for regret that cases of small-pox and infectious fevers are treated in rooms situated above the accident ward. It is not justifiable, under any circumstances, to treat infectious disease in wards situated in the main building of a hospital, the major portion of which is utilised for the treatment of cases of general disease. Of course, a relatively young city like Winnipeg cannot attain maturity in a month, or even in ten years, but it has done so much for its hospital that the municipality may reasonably be expected to come to the rescue of the General Hospital authorities, and to take immediate steps to open an infectious hospital in an isolated situation, quite apart from all other institutions.

We visited the hospital on Sunday, and found the patients surrounded by their friends, who seem to go in great numbers, and without any limit. We understood that a hundred and fifty visitors were there in the course of the day, and, as some of the patients in the general wards were very ill, we are of opinion that steps should be taken to limit the number of friends admitted to each patient, and to fix regular visiting days, as is done in the old country.

The fittings throughout the buildings are kept in good repair. The baths are of polished metal, and have been recently renewed, together with the w.c.'s, slop sinks, &c. We have no wish to be unreasonable, but, as the Medical Officer in his report states that the sanitary arrangements of the hospital are in excellent condition, we think it necessary to point out that all the waste-pipes should empty outside the hospital over an open gully trap, and so have no direct communication with the sewers. Similarly, the soil pipes should be disconnected from the sewers by an open man-hole and syphon-trap placed between the sewer and the hospital. Until these two things are done, it is at least incorrect—if not dangerous—to describe the sanitary arrangements as excellent.

### The Nurses' Home.

The Nurses' Home consists of a separate house, and presents a pleasant appearance externally. It is connected with the hospital building by the basement. The nurses have a separate sitting-room, which is comfortably furnished. The library contained little except handsomely-bound copies of *The Queen* newspaper, which seemed to be highly prized in Winnipeg. We imagine that *The Queen* has never been so handsomely encased in its previous history, and that, much as the paper is valued by many people, it has never before been promoted to the high position of pre-eminence it occupies at Winnipeg.

The staff of nurses consists of a Head Nurse and four trained nurses, in addition to the Matron—two of the former having graduated from the Winnipeg Training School. Twelve pupil nurses are undergoing instruction at the present time. Nurses are supplied to the outside public by the hospital authorities—61 cases having been attended at private houses out of 120 applications during 1890. Applicants have to communicate with the Medical Superintendent, who is practically master of the Nurses' Training School. If approved after a period of two months' probation—during which time applicants receive board and lodging at the expense of the hospital—pupil nurses are admitted to a course of two years' training, and receive six dols. (24s.) per month the first six months; seven dols. (28s.) the second six months; and 10 dols. (£2) a-month for the last year. Pupil nurses are obliged to serve either as nurses in the hospital, or to attend to private cases among the rich and poor in any part of the Province of Manitoba. This outside nursing is liable to lead to serious abuses, and we should hope that it will be discontinued in the case of all pupil nurses of less than a year's standing, at any rate, so soon as the school is fully organised. The school is non-sectarian, and courses of lectures with practical instruction are given in the wards, and at the bedside, by the Medical Superintendent.

The pupil nurses have to pass a final examination in the studies of each course in April, or at the completion of their two years' course. Those nurses who are successful receive diplomas certifying that they have gone through the course, and are duly qualified nurses.

There seems to be a dual control, well calculated to lead to mischief, as the medical superintendent has the power to suspend a pupil nurse or nurses for negligence or misconduct while on duty; the same power being vested in the matron during the time these members of the staff are off duty.

### The Maternity Hospital.

The Maternity Hospital is a small separate and partly isolated house, and has done good service in many cases. Many of the farmers' wives from the prairie resort to it to be confined, and, so far, there has not been a single fatal case. This branch of the work is spoken of as being of the utmost value to the people in the North-West, provided care is taken to prevent the usual dangers which have so often brought about the closing of similar institutions in the old country. The medical superintendent was so courteous and kind, so anxious to show us everything, that we are indisposed to criticise. Still, no useful purpose can be served by withholding the opinion that there is an absence of discipline throughout the whole hospital, which militates against its efficiency and general appearance. We are convinced that it is desirable in the best interests of all those who take an interest in the institution, and especially of those who enter it as patients, that the system of administration should be so modified as to secure that one person of capacity and knowledge, and one only, shall be entrusted with the whole administration of the establishment.

As we read the rules, and as we gather from some conversation we had with the residents in the town, there is at present a dual control in more than one department of the hospital, and it is a matter for regret that the largest hospital in the North-West Provinces should not present a more efficient and cleaner appearance, and one which would prove to the public that all was well within its walls. A few more years of the present system, or rather want of system, will make the Winnipeg General Hospital a bye-word and a reproach throughout the North-West.

Medicine Hat.

This is a delightful little hospital, well situated and well planned in every way. It owes its foundation to the energy and devotedness of Mr. J. Niblock, the President, and it would be well for all the hospitals of the world if each one of them possessed for its president a gentleman of the energy and capacity of Mr. Niblock.

The hospital consists of (*a*) four large wards, two for men and two for women, which are devoted to medical and surgical cases; (*b*) four small wards for incurable cases; and (*c*) a maternity department containing about six beds. The whole institution impresses one favourably, everything is beautifully clean and well arranged. Miss Reynolds, the Matron, who took us round, was trained at Leeds, and certainly does the hospital and her parent school great credit. All the patients were cheerful and well cared for. The nurses have their own separate sitting-room and dining-room, and they each have a separate bed-room also. If we wanted to briefly prove the great efficiency of the management of this hospital, we would ask any gentleman to visit the incurable wards. There are two cases of paralysis, both men; one—an old man—very difficult to manage. The condition of these cases, their cleanliness, cheerfulness, and the whole aspect of the wards in which they were lying, proved that Miss Reynolds and her assistants are not only efficient, but devoted, women. In the Maternity Department was a farmer's wife, who had just presented her husband with twins. Of these twins, one was asleep, the other awake, at the time of our visit. The mother seemed distressed lest they should form a habit of sleeping alternately, and so prevent her and their father from getting necessary rest! We understood that the latter was delighted, but the poor mother, quite justly, as we think, was evidently anxious as to how far she would be able to manage two babies at one time in the limited space at her disposal in the log-house at home. We noticed that newspapers and books, and even a few pictures, would prove very acceptable, and we hope that some readers of The Hospital will place themselves in communication with Miss Reynolds, Medicine Hat Hospital, North-West Territory, and so keep her well supplied with all that she may require, and with other small comforts that she may be able to mention to them. We congratulate Mr. Niblock and all concerned in the management of the Medicine Hat General Hospital upon its efficiency, cleanliness, good order, and arrangements. In all these respects it is in striking contrast to the hospital at Winnipeg—a fact as creditable to Medicine Hat as it is surprising to the visitor. It is further interesting to note that Miss Reynolds trains nurses, with the aid of the medical staff, and so may be regarded as the Superintendent of the "highest" nurse training school probably in the whole world—Medicine Hat being 2,150 feet above the level of the sea. An arrangement had been made by the railway officials not to start the train until your Commissioner returned from the hospital. The conductor of the train, however, forgot all about these instructions, and so went off with the train before we got back to the station. There was no hotel, no dinner, and no other train for twenty-four hours. If the departed express could be caught, your Commissioner would secure dinner, bed, and transit. He therefore chartered an engine, set off in pursuit, and after an exciting chase caught the express at the end of a fast run of 35 miles.

